# Hard-to-treat or hard-to-catch? Clinical features and therapeutic outcomes of help-seeking foster care youths with mood disorders

**DOI:** 10.3389/fpsyt.2023.1211516

**Published:** 2023-10-13

**Authors:** Xavier Benarous, Hélène Lahaye, Hugues Pellerin, Angèle Consoli, David Cohen, Réal Labelle, Johanne Renaud, Priscille Gérardin, Fabienne El-Khoury, Judith van der Waerden, Jean-Marc Guilé

**Affiliations:** ^1^Department of Child and Adolescent Psychopathology, Amiens University Hospital, Amiens, France; ^2^INSERM Unit U1105 Research Group for Analysis of the Multimodal Cerebral Function, University of Picardy Jules Verne (UPJV), Amiens, France; ^3^Department of Child and Adolescent Psychiatry, Pitié-Salpêtrière University Hospital, APHP, Paris, France; ^4^CNRS UMR 7222, Institute for Intelligent Systems and Robotics, Sorbonne University, Paris, France; ^5^Department of Psychology, Université du Québec à Montréal, Montréal, QC, Canada; ^6^Centre for Research and Intervention on Suicide, Ethical Issues and End-of-Life Practices, Université du Québec à Montréal, Montréal, QC, Canada; ^7^Manulife Centre for Breakthroughs in Teen Depression and Suicide Prevention, Douglas Mental Health University Institute, McGill University, Montréal, QC, Canada; ^8^Department of Psychiatry, McGill University, Montréal, QC, Canada; ^9^Department of Child and Adolescent Psychopathology, Rouen University Hospital, Rouen, France; ^10^Sorbonne Université, INSERM, Institut Pierre Louis d’Epidémiologie et de Santé Publique, Equipe de Recherche en Epidémiologie Sociale, Paris, France; ^11^Pôle de psychiatrie de l’enfant et de l’adolescent, Etablissement Publique de Santé Mentale de la Somme, Paris, France

**Keywords:** depressive disorder, early-onset bipolar disorder, disruptive mood dysregulation disorder, child adversity, complex psychotrauma, developmental psychotrauma, adverse chilhood experiences

## Abstract

**Introduction:**

The high level of emotional problems in youths placed in foster care contrasts with the limited use of evidence-based treatments. This study aims to better characterize the clinical features and therapeutic outcomes of foster care youths with mood disorders.

**Methods:**

A secondary analysis of data collected in the context of a French-Canadian clinical research network on pediatric mood disorders in four sites was conducted to compare three groups of patients with depressive or bipolar disorder: those without exposure to child welfare intervention (WCWI, *n* = 181), those who received non-placement psychosocial intervention (NPI, *n* = 62), and those in placement interventions (PI, *n* = 41).

**Results:**

We observed a very high rate of academic problems in patients in the groups NPI/PI compared to those in the WCWI group. Patients in the PI group had more disruptive behavioral disorders (OR = 6.87, 95% CI [3.25–14.52]), trauma-related disorders (OR = 3.78, 95% CI [1.6–8.94]), and any neurodevelopmental disorders (OR = 2.73, 95% CI [1.36–5.49]) compared to the other groups (NPI/WCWI). Among inpatients, the Clinical Global Impression-Improvement scale and the change in the Children Global Assessment Scale during the hospital stay did not differ across the three groups. We observed a higher prescription rate of antipsychotics in the PI group compared to the NPI/WCWI groups, but no significant difference for antidepressants and mood stabilizers.

**Discussion:**

These findings support the view that, when provided with dedicated support, fostered inpatient youths can improve in a range comparable to other inpatients. Undetected neurodevelopmental disorders and academic problems are likely important contributors of the burden of mood disorders in these youths.

## 1. Introduction

### 1.1. Mental health difficulties in foster care

Young people in the foster care system are identified as a high-risk group for emotional and behavioral problems as well as psychiatric disorders (1, 2). These youths are especially prone to trauma and stressors-related disorders, disruptive behavioral disorders, substance abuse, and suicidal behaviors (1, 2). The global mental health burden of youths in foster care stems from multiple factors including exposure to repeated adverse childhood experiences (ACEs) (including the ones which led to the placement decision), abrupt life transitions, and associated environmental and neurodevelopmental factors (3–6).

The evaluation of the effectiveness of mental health interventions in youths in foster care received surprisingly little scrutiny considering the number of patients concerned. Several reports note that youths in foster care are over-represented in pediatric mental health services, with almost 25–53% of them having already been in contact with mental health professionals (7–9). Yet, the mental health care provided to these patients is usually regarded as suboptimal by health professionals with a high use of emergency services and a low access to evidence-based interventions targeting emotional and trauma-related symptoms (5, 6, 10) and learning disability if any (4).

### 1.2. Clinical presentations of mood disorder in youths in foster care

Foster care patients with mood disorders are expected to present more severe forms of mood disturbances compared to those without exposure to child welfare intervention, considering the very high rate of major ACEs reported in this population (6). The exposure to major ACEs (i.e., physical, sexual, emotional abuse and/or physical, emotional neglect) is correlated with more severe depressive symptoms, more severe suicidal behaviors, more frequent psychotic symptoms, earlier age of onset, and more recurrent and chronic course of the disorder with a decreased rate of remission (11–14). Comparable trends have also been observed in adults with bipolar disorder (BD) where a history of major ACEs is associated with more frequent suicidal behaviors and more frequent psychotic and mixed features (15).

The clinical challenges of assessing mood symptoms in pediatric patients may be particularly exacerbated in patients in foster care (16, 17). Among the most common pitfalls, youths with trauma-related symptoms may exhibit manic-like symptoms (e.g., emotional lability, behavioral disinhibition) or ADHD-like symptoms (e.g., increased distractibility, hyperkinetic) in response to acute stressors (18–20). A large overlap exists between symptoms of post-traumatic and depressive disorders, such as self-injurious behaviors in response to trauma-related stimuli, social withdrawal, and impaired sleep (19). Finally, even in the absence of post-traumatic symptoms, a history of life trauma may influence a youth’s ability to trust and share concerns and emotional experiences with mental health professionals (21).

The identification of mood symptoms in foster care youths is also important as it may influence placement prognosis. Longitudinal studies have shown that the level of a youth’s mood symptoms at admission into the welfare system often predicts the onset of behavioral problems in foster care (22–24). This is an issue of particular significance for careers considering the interplay between foster care youth’s emotional and behavioral problems and placement instability (22–24). Anderson (22) noted that the likelihood of suicidal ideation increased by 68% each time a child experienced a change in placement situation. Effective interventions on mood symptoms in this population are therefore opportunities to break this vicious circle (23, 24).

### 1.3. Therapeutic outcomes of mood disorder in youths in foster care

While childhood maltreatment has been found to be associated with a lower probability of response to antidepressant pharmacotherapy and psychotherapy in depressed (14) and bipolar adults (25), these findings have not been replicated in pediatric samples. Secondary analyses of data from clinical trials of antidepressants have shown that a history of sexual violence did not significantly influence the response rate of provided medications in children and adolescents (26–29). ACEs status did not influence the average response to mood stabilizers during the acute treatment of manic or mixed episodes in 81 adolescents in the study conducted by Benarous et al. (30).

Discrepancy also exists regarding how ACEs status could influence the functional improvement during the hospital stay of inpatient adolescents with mood disorders. In a chart-review with over 10 years of follow-up, Serim Demirgoren, Ozbek, and Gencer (31) noted that high familial risk scores at admissions are associated with lower functional improvement during the stay of 308 Turkish children and adolescents. However, the exposure to ACEs or child welfare interventions did not influence the average change in CGAS, the average Clinical Global Impression-Improvement scale, or the average length of stay in 106 adolescents hospitalized for severe or treatment-refractory mood disorders (32). Some authors have even suggested that youths exposed to ACEs or child welfare interventions could benefit more in terms of their general functioning during inpatient treatment compared to other patients as they are removed from potential ongoing stressors (31, 33).

While these findings could be mitigated when considering the impact of ACEs on the therapeutic outcomes of psychotherapies (34), they suggest that mood disorders should not be left untreated even in patients who are usually regarded as “complex.” In this vein, the American Academy of Pediatrics recommends that the general guideline for the use of antidepressant should apply to youths indistinctly of the context of exposition to childhood maltreatment (5).

### 1.4. Aims

This research is a secondary analysis of data initially collected in three observational cross-sectional studies conducted within a French-Canadian clinical research network on pediatric mood disorder and suicidal behavior. These researches aimed to test a series of hypotheses on the clinical features and therapeutic outcomes of different subtypes of pediatric mood disorders, in particular the category of Disruptive Mood Dysregulation Disorder (DMDD). Here, we aim to compare the clinical features and therapeutic outcomes of patients with mood disorders in three groups of patients: those without exposure to child welfare intervention (WCWI), those in non-placement psychosocial intervention (NPI), and those in out-of-home placement interventions (PI). The comparison between youths in the NPI and in the PI groups would help to better distinguish the effect of risk factors related to placement (involving family separation) from the effect of co-occurring environmental risk factors (i.e., demographic, perinatal, and psychosocial) (3, 6). It is indeed worth remembering that the possible separations from the youth’s close relationships (e.g., siblings, grand-parents, friends, teachers) in out-of-home placements could result in the loss of protective factors for mood disorders (35).

Regarding clinical outcomes, we tested whether patients with mood disorders in foster care present on average a higher severity of mood symptoms and a poorer level of functioning compared to those WCWI, in line with the literature about ACEs (11, 12, 14) and foster care patients (16, 17). We also hypothesized that patients in the NPI group would have an “intermediate” severity profile compared to the two other groups, and that a higher rate of school problems would be observed in youths in PI compared to those in NPI and WCWI, in line with previous findings (36, 37). Patients with mood disorders in the PI group were expected to present higher comorbidity rates of psychiatric and neurodevelopmental disorders compared to patients from the two other groups. This would be consistent with previous reports stressing the importance of perinatal factors in this population and the high rate of neurodevelopmental disorders generally reported in PI youths (1).

Regarding therapeutic outcomes, we expect that the treatment provided for pediatric mood disorders would differ between inpatients in the PI, NPI, and WCWI groups in line with the previous studies conducted in youths with ACEs mentioned above (4, 5, 10). A higher rate of antipsychotic treatment is to be expected in the PI group as well as a lower access to specific treatment (i.e., antidepressants, and mood stabilizers) (5). No *a priori* hypotheses were made regarding the clinical and functional effectiveness of the treatment provided for inpatient adolescents with mood disorders considering the discrepant reports in the literature mentioned above (30–32).

## 2. Methods

### 2.1. Settings and study design

This research consists of a secondary analysis of data collected in three observational cross-sectional studies conduct in the framework of a university French-Canadian clinical research network on pediatric mood disorders and suicidal behaviors. The research network was developed in view of studying in view of studying the specific clinical features and predictors of treatment response of youths with pediatric mood disorders and/or severe suicidality to guide policy decisions and preventive strategies. The context for the hospitalization and the main intervention provided were previously detailed in published papers (32, 38–40).

- Site 1: inpatients referred to two adolescent inpatient units (for 12- to 15 years-old and for 15- to 18 years-old patients, with 30 beds) at the Pitié-Salpêtrière Hospital, Paris, France, between January 2017 and December 2018.
- Site 2: inpatients referred to pediatrician-psychiatric crisis-center inpatient unit with 12 beds at the Amiens University Hospital, France, between February 2020 and April 2021.
- Site 3: outpatients referred to psychiatric outpatient unit specialized in pediatric mood disorders at the Rivière des Prairies Hospital, Montréal, Canada, between November 2006 and December 2010
- Site 4: outpatients referred to psychiatric outpatient unit specialized in pediatric mood disorders at the Douglas Mental Health University Institute, Montréal, Canada, between November 2006 and December 2010.

Prior authorization and approval from independent ethics committee were previously received from each competent local authority in line with national legislation as presented in previous studies, i.e., for site 1, for site 2, for sites 3–4. No new data was collected for the current research.

### 2.2. Participants

**Figure 1:**
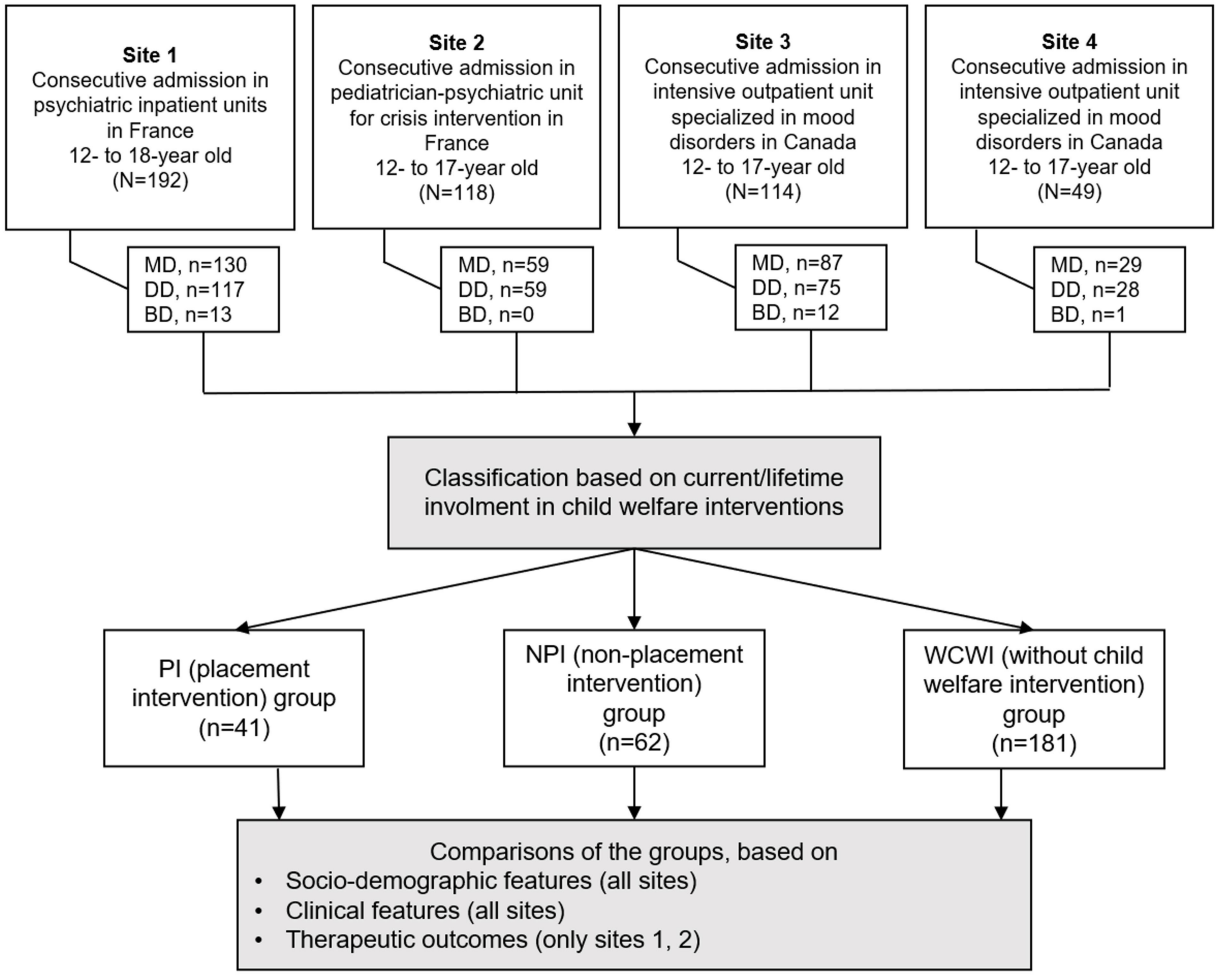
Flow chart of the study. MD, mood disorders; DD, depressive disorders; BD, bipolar disorders.

For this study, we extracted information from patients with a discharge psychiatric diagnosis of mood disorders. The psychiatric diagnoses had been defined according to the Diagnostic and Statistical Manual of Mental Disorders, 5th edition (DSM-5) categories (American Psychiatric Association 2013); that is, a major depressive disorder (MDD), a persistent depressive disorder (PDD), a DMDD, and BD. No exclusion criteria were used. The flow chart presents the selection of participants (Figure 1). Taking into account the debate about the wide spectrum of pediatric bipolar disorder (41), only type-I bipolar disorder was included in the BD category.

In France and in Canada placement in foster care is decided when that child is no longer able to live safely at home. A court grants legal guardianship for the child to the state, and Child Protective Services (*Aide Social à l’Enfance* in France, *Direction de la protection de la jeunesse* in Canada) is subsequently granted temporary legal possession to place the child in foster care. The decision can made in an emergency or after a period when non-placement psychosocial interventions had been provided to the family.

For all participants, child welfare interventions were systematically sought during a clinical interview with the child, parent, health professionals and, if needed a social worker in line with previous studies in youths with mood disorders (4, 42, 43). We distinguished between PI and NPI. PI encompassed out-of-home psychosocial interventions, such as short-term shelter, foster home, rural living facility, and foster family. NPI also included in-home psychosocial intervention usually required by judiciary and legal services to investigate suspicion of maltreatment. Patients referred to the hospital by administrative or legal authorities for immediate protection or when parental consent was not received were classified as PI. The classification was based on current or lifetime prior involvement in the child welfare system. For example, a 12 years old child who lived in a short-term shelter during 6 months had at 8 years old been included in the PI group. Participants in the WCWI group represented patients who had never been involved in psychosocial interventions (placement or non-placement) in child welfare system.

Participants were classified as having experienced “maltreatment” if they were ever exposed to at least one form of major ACEs, i.e., physical abuse, sexual abuse, emotional abuse and/or severe physical or emotional neglect. The category was rated by the clinicians involved in the patient’s care based on all information available. The external validity of this measure compared to information based on social services evaluations was empirically supported in a previous study (30, 44). Information about the type of maltreatment was only available for sexual abuse. No other information was available concerning the age at onset, frequency, the duration or the setting of experienced major ACEs, in particular the involvement of other family members and the contribution to the placement decision.

### 2.3. Assessments

The data collected encompassed sociodemographic characteristics; school performance; psychosocial factors; developmental history and associated medical conditions; clinical characteristics, including the discharge psychiatric diagnoses; symptom severity, level of functioning, and treatment response.

#### 2.3.1. Clinical features and associated disorders

The discharge psychiatric diagnoses were selected among a list of the most frequently used categories. The Schedule for Affective Disorders and Schizophrenia for School-Age Children PL (K-SADS-PL) (45) was included in the clinical assessment at the sites 3 and 4, but only in case of uncertainty in the sites 1 and 2. In these cases, the psychiatric diagnoses retained were the ones made by treating clinicians based on all information available. As this instrument refers to the DSM-IV-TR classification the following changes have been made. All participants meeting criteria for dysthymia have been identified as PDD. Concerning the DMDD diagnosis, symptoms reported in the patient’s medical file were compared with the DSM-5 criteria for DMDD. The psychometric properties of this retrospective assessment was estimated to be very good (Cronbach’s *α* for internal validity: 0.90; *κ* for test–retest reliability: 0.87) (41).

The severity of clinical symptoms was measured with the Clinical Global Impression-Severity (CGI-S) scale (46), and the level of functioning was assessed using the Children-Global Assessment Scale (CGAS) (47). The CGI-S and the CGAS were only available in sites 1 and 2 as it was not used in routine practice at Canadian sites. The CGAS was systematically measured the first and the last week of stay by a senior psychiatrist (respectively, “CGAS admission” and “CGAS discharge”).

#### 2.3.2. Therapeutic outcomes in inpatients (site 1 and 2)

Classes of psychopharmacological treatments prescribed at discharge were classified into five categories (i.e., anxiolytics, hypnotics, antidepressants, mood stabilizers, antipsychotics). Therapeutic outcomes were assessed using two proxy measures available for sites 1 and 2. Clinical improvement during the hospitalization was measured with the Clinical Global Impression-Improvement (CGI-I) scale (46), completed by a senior psychiatrist during the last week of the hospitalization. In this study, the CGI-I assessed the patient’s overall symptom improvement during the hospitalization compared with his/her baseline state at admission, irrespective of the treatments provided (e.g., medication, structured psychotherapy, group interventions). We used the difference between the CGAS score at discharge and at admission, also labeled Δ-CGAS to track change in the level of functioning during the hospitalization. Unfortunately, information was not available for the Canadian sites.

### 2.4. Statistical analysis

Continuous variables (e.g., age) were described using mean and standard deviation; categorial variables (e.g., gender) using the number and percentage of occurrences. Three groups were compared: those in the WCWI group; those in the NPI group, those in the PI group. Analysis of variance (ANOVA) was used to compare means across the three groups. *Post hoc* Scheffe’s tests were used for the comparisons between groups when ANOVA yielded a significant *F*-statistic. Chi-squared test was used to compare proportions across the three groups. The Kruskal–Wallis and the Fisher’s exact tests were used as alternatives for non-normally distributed variables. No mathematical correction was made for multiple comparisons. The listwise deletion was used for missing values. Data were analyzed using R.

## 3. Results

### 3.1. Socio-demographic features and school functioning

There was no statistical difference in the three groups concerning mean age and gender (Table 1). Subjects in the PI and the NPI groups reported a higher rate of maltreatment compared to patients in the WCWI group (respectively, OR = 13.40, 95% CI [5.99–29.96] and OR = 6.40, 95% CI [3.41–12.01]). The likelihood of being exposed to sexual abuse was most important in the PI group, followed by those in the NPI, followed by the WCWI (PI vs. WCWI, OR = 14.77, 95% CI [6.10–35.79]).

**Table 1:** Sociodemographic features of youths with mood disorders in placement intervention vs. non-placement intervention vs. without child welfare intervention.

	Subjects without child welfare intervention (*n* = 181)	Subjects with child welfare intervention		*p* value
		Non-placement intervention (*n* = 62)	Placement intervention (*n* = 41)	
Demographic features				
Gender, female	76 (42%)	31(50%)	19 (43%)	0.528
Age (*y*) (mean ± SD)	14.61 ± 2.16	14.18 ± 2.41	14.22 ± 1.56	0.271
SES, good and middle, *n* (%)	164 (91%)^a^	40 (65%)^b^	18 (44%)^c^	<0.001^**^
Psychosocial factors				
Maltreatment, all types, *n* (%)	34 (19%)^a^	37 (60%)^b^	31 (76%)^b^	<0.001^**^
Sexual abuse, *n* (%)	10 (6%)^a^	13 (21%)^b^	19 (46%)^c^	<0.001^**^
School performance				
Grade repetition, *n* (%)	25 (14%)^a^	12 (20%)^ab^	14 (36%)^b^	0.006
Special educational needs, *n* (%)	48 (28%)^a^	21 (36%)^ab^	22 (58%)^b^	<0.001^**^
School dropout (>3 months), *n* (%)	47 (26%)^a^	23 (37%)^a^	26 (65%)^b^	<0.001^**^
Special educational facilities, *n* (%)	5 (3%)^a^	10 (17%)^b^	7 (17%)^b^	<0.001^**^

^*^indicates *p* < 0.05 and ^**^indicates *p* < 0.01. ^a–c^Means in a row without a common superscript letter differ (*p* < 0.05) in post hoc analyses. SES, socio-economic status.

Compared to patients in the WCWI group, adolescents with mood disorders in the PI group had on average higher rates of grade repetitions (OR = 3.24, 95% CI [1.50–7.01]), of special educational needs (OR = 3.21, 95% CI [1.60–6.44]), of school dropouts (OR = 4.94, 95% CI [2.41–10.12]) and referral to special educational facilities (OR = 7.25, 95% CI [2.17–24.19]). Differences between patients’ characteristics in the PI and the NPI groups were only significant for school dropouts.

### 3.2. Aim 1: clinical features

The proportion of each subtype of depressive disorders differed across the three groups (Table 2). In the PI group, DMDD was the most frequent depressive subtype, followed by PDD and then MDD. In contrast, in the WCWI group, MDD was the most frequent depressive subtype, followed by PDD and then DMDD. Youths in NPI had an intermediate profile. Youths in PI and in NPI groups were more likely to have chronic irritability compared to youths in the WCWI (respectively OR = 3.25, 95% CI [1.63–5.46]; OR = 3.41, 95% CI [1.85–6.28]). Youths in PI were more likely to have repeated runaway and substance misuse compared to the two other groups (vs. WCWI group, respectively, OR = 13.38, 95% CI [5.51–32.49]; vs. NPI OR = 6.00, 95% CI [2.52–14.27]). The likelihood of suicidal ideation, suicidal attempt, non-suicidal self-injury, and psychotic symptoms did not significantly differ across groups. The mean CGAS score and CGI-S score at admission were not statistically significant across groups in inpatient adolescents (sites 1 and 2).

**Table 2:** Clinical features of mood disorders among youths in placement intervention vs. non-placement intervention vs. without child welfare intervention.

	Subjects without child welfare intervention (*n* = 181)	Subjects with child welfare intervention		*p* value
		Non-placement intervention (*n* = 62)	Placement intervention (*n* = 41)	
Types of mood disorders, *n* (%)				
MDD	81 (45%)^a^	20 (32%)^a^	5 (12%)^b^	<0.001^**^
PDD	67 (37%)	24 (39%)	19 (46%)	0.542
DMDD	40 (22%)^a^	23 (37%)^b^	24 (59%)^c^	<0.001^**^
BD-I	12 (7%)	7 (11%)	5 (12%)	0.282
Clinical characteristics, *n* (%)				
SI	132 (73%)	45 (73%)	27 (66%)	0.654
SA	57 (32%)	23 (37%)	16 (39%)	0.540
NNSI	78 (43%)	22 (36%)	20 (49%)	0.375
Psychotic symptoms	21 (12%)	14 (23%)	8 (20%)	0.084
Chronic irritability	69 (38%)^a^	42 (68%)^b^	28 (68%)^b^	<0.001^**^
Repeated runaway	10 (9%)^a^	9 (24%)^b^	18 (51%)^c^	<0.001^**^
Substance misuse	13 (19%)^a^	5 (16%)^a^	13 (43%)^b^	0.015^*^
Clinical severity and functioning in inpatient adolescents (site 1 and 2)				
CGAS at admission (mean ± SD)	41.29 ± 11.3	39.21 ± 13.56	38.03 ± 13.96	0.378
CGI-S at admission (mean ± SD)	4.94 ± 0.96	4.95 ± 1.01	5.23 ± 0.97	0.295

^*^indicates *p* < 0.05 and ^**^indicates *p* < 0.01. ^a–c^Means in a row without a common superscript letter differ (*p* < 0.05). MDD, major depressive disorder; PDD, persistent depressive disorder; DMDD, disruptive mood dysregulation disorder; BD, bipolar disorder; SI, suicidal ideation; SA, suicidal attempt; NNSI, non-suicidal self-injury; CGAS, children-global assessment scale; CGI-S, clinical global impressions-severity.

The number of psychiatric diagnoses at discharge was more frequent in patients in the PI group (M = 2.44, SE = 1.05) and in the NPI group (M = 2.32, SE = 1.08) compared to those in the WCWI group (M = 1.88, SE = 1.00), *p* = 0.001; while the difference between the PI and the NPI did not reach statistical significance (*p* = 0.572) (Table 3). Compared to youths in the WCWI group, those in the PI group were more likely to have associated disruptive behavioral disorders (OR = 6.87, 95% CI [3.25–14.52]) and trauma-related disorders (OR = 3.78, 95% CI [1.6–8.94]) but were less likely to have anxiety disorders (OR = 0.44, 95% CI [0.18–1.05]). The comorbidity rate did not significantly differ between youths in the PI and NPI groups.

**Table 3:** Associated psychiatric, developmental, and medical conditions in youths with mood disorders in placement intervention vs. non-placement intervention vs. without child welfare intervention.

	Subjects without child welfare intervention (*n* = 181)	Subjects with child welfare intervention		*p* value
		Non-placement intervention (*n* = 62)	Placement intervention (*n* = 41)	
Associated psychiatric disorders, *n* (%)				
Anxiety disorders	58 (32%)^a^	11 (18%)^ab^	7 (17%)^b^	0.027^*^
Trauma- & stressor-related disorders	16 (9%)^a^	9 (15%)^ab^	11 (27%)^b^	0.007^*^
Disruptive behavioral disorders	24 (13%)^a^	27 (45%)^b^	21 (51%)^b^	<0.001^**^
Psychotic disorders	3 (2%)	1 (2%)	1 (2%)	0.820
Eating disorders	14 (8%)	3 (5%)	1 (2%)	0.491
Associated neurodevelopmental disorders, *n* (%)				
Attention deficit disorder	23 (13%)^a^	18 (29%)^b^	6 (15%)^ab^	0.011^*^
Autism spectrum disorder	5 (4%)	1 (3%)	1 (3%)	0.999
Intellectual development disorder	2 (2%)^a^	5 (13%)^b^	4 (11%)^b^	0.006*
Communication disorder	7 (6%)^a^	9 (24%)^b^	9 (28%)^b^	<0.001^**^
Developmental coordination disorder	22 (20%)^a^	13 (35%)^b^	12 (39%)^b^	0.045^*^
Specific learning disabilities	10 (9%)^a^	10 (26%)^b^	6 (19%)^ab^	0.024^*^
Other developmental difficulties				
Pregnancy complications/fetal distress, *n* (%)	10 (9%)^a^	6 (17%)^ab^	10 (36%)^b^	0.003^*^
Speech acquisition delay, *n* (%)	14 (13%)^a^	13 (34%)^b^	13 (39%)^b^	0.001^*^
Motor acquisition delay, *n* (%)	21 (19%)^a^	15 (40%)^b^	15 (47%)^b^	0.003^**^
Medical conditions				
Chronic medical condition, *n* (%)	58 (32%)^a^	26 (42%)^ab^	22 (54%)^b^	0.027^*^
Overweight, *n* (%)	17 (15%)	12 (32%)	8 (23%)	0.073

^*^indicates *p* < 0.05 and ^**^indicates *p* < 0.01. ^a–c^Means in a row without a common superscript letter differ (*p* < 0.05) in post hoc analyses.

The number of neurodevelopmental disorders at discharge was more frequent in patients in the PI group (M = 1.03, SE = 1.21) and in the NPI group (M = 1.03, SE = 1.21) compared to those in the WCWI group (M = 0.41, SE = 0.71), *p* = 0.001; while the difference between the PI and the NPI did not reach statistical significance (*p* = 0.904). Compared to youths in the WCWI group, those in the PI were more likely to receive a diagnosis of associated neurodevelopmental disorder (53% vs. 31%, OR = 2.73, 95% CI [1.36–5.49], *p* = 0.023), without difference with the NPI group (51%). Youths in the PI were more likely to have a diagnosis of intellectual developmental disorder (OR = 10.23, 95% CI [1.80–58.03]), communication disorder (OR = 6.99, 95% CI [2.43–20.12]) or developmental coordination disorder (OR = 2.99, 95% CI [1.33–6.70]) compared to youths in the WCWI group. Greater delays in motor and language acquisitions existed in young people in PI compared to those in the WCWI group.

On average, patients in the PI group were the most likely to have prior hospitalization (any and repeated), an admission via the emergency room, and constraints during the stay (Table 4). The rate of prior contact with a psychologist, psychiatrist, and speech interventions did not differ across the three groups. Occupational therapy was more frequently observed in patients in the NPI group compared to the other two groups.

**Table 4:** Mental health service use of youths with mood disorders in placement intervention vs. non-placement intervention vs. without child welfare intervention.

	Subjects without child welfare intervention (*n* = 181)	Subjects with child welfare intervention		*p* value
		Non-placement intervention (*n* = 62)	Placement intervention (*n* = 41)	
Mental health service use, *n* (%)				
Prior hospitalization	59 (33%)^a^	25 (40%)^a^	32 (78%)^b^	<0.001^**^
Prior multiple hospitalizations	26 (14%)^a^	14 (23%)^a^	22 (54%)^b^	<0.001^**^
Admission via ER	59 (33%)^a^	20 (32%)^a^	27 (66%)^b^	<0.001^**^
Constraint measures during the stay	8 (7%)^a^	7 (18%)^b^	18 (51%)^c^	<0.001^**^
Speech therapy	19 (17%)	11 (29%)	9 (26%)	0.211
Occupational therapy	18 (16%)^a^	12 (32%)^b^	4 (11%)^a^	0.049^*^

^*^indicates *p* < 0.05 and ^**^indicates *p* < 0.01. ^a–c^Means in a row without a common superscript letter differ (*p* < 0.05).

### 3.3. Aim 2: therapeutic outcomes in inpatients (sites 1 and 2)

Among adolescent inpatients (sites 1 and 2), the number of medications in patients in the PI group (M = 2.10, SE = 1.57) was overall not statistically different (*p* = 0.96) from patients in the NPI group (M = 1.59, SE = 1.12) or those in the WCWI group (M = 1.40, SE = 1.35). Youths in the PI were more likely to receive an antipsychotic medication compared to youths in the WCWI groups (59% vs. 31%, OR = 3.15, 95% CI [1.57–6.32], *p* = 0.009), without significant difference with the NPI group (49%, *p* = 0.188). The rate of antidepressant treatment did not statistically differ between patients in the PI, NPI and WCWI groups (respectively, 31, 28 34%, *p* = 0.799) nor did the rate of mood stabilizers (respectively, 13, 13 12%, *p* = 0.999).

**Figure 2:**
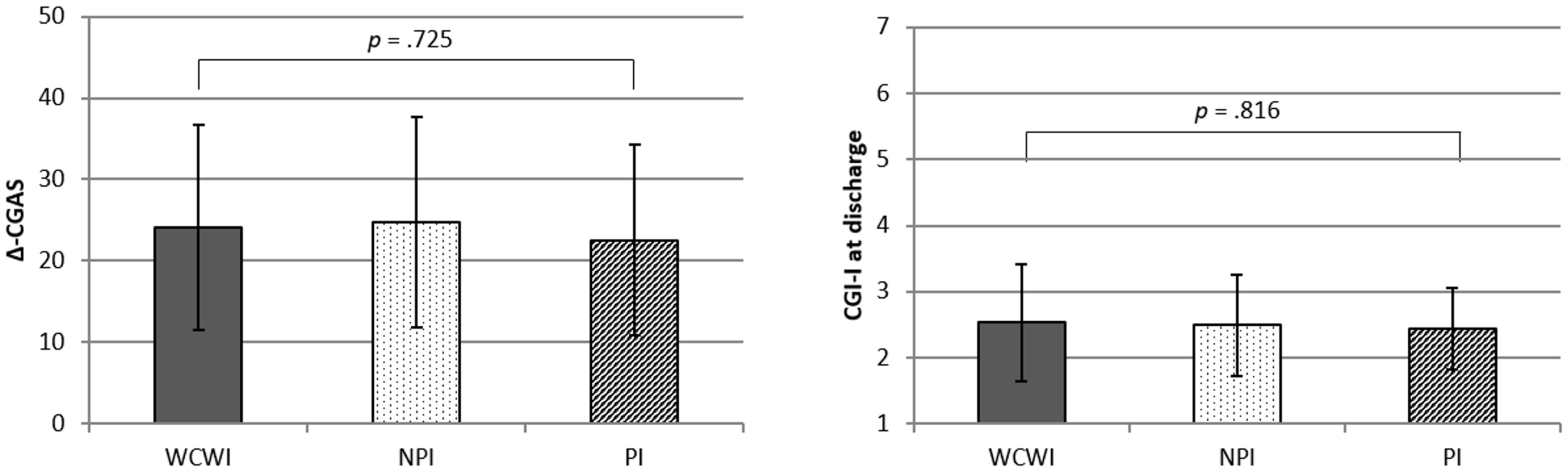
Comparisons between the average levels of patients’ clinical and functioning improvement between the groups.

As shown in Figure 2, no significant difference was observed across the three groups concerning the CGI-I score at discharge and the Δ-CGAS score among inpatient adolescents (site 1 and 2). Based on the CGI-I score 56% of patients in the PI were found to be well or very well improved during the stay, vs. 46% among subjects in the NPI group and vs. 56% in the WCWI group (*p* = 0.578).

## 4. Discussion

### 4.1. Summary of the main results

This study aimed to describe the clinical features and the therapeutic outcomes of foster care youths with mood disorders compared to their counterparts without child welfare intervention. This research was motivated by the lack of information to guide care decisions for foster care youths, despite their frequent use of pediatric mental health care facilities in particular emergency services in most developed countries (7–9).

#### 4.1.1. Clinical features

On average, patients in the PI group more frequently had chronic forms of depressive disorders, such as DMDD, and reported chronic irritability. These results are consistent with prior studies stressing a relation between ACEs and chronic irritability (48). The translational model of irritability developed by Brotman et al. (49) highlights the influence of parental roles in the development of the child’s emotional regulation skills. In a context of foster care, dysfunctional early parent–child interactions, such as chaotic and unpredictable parental reactions to a child’s emotional expression, could minimize the process of developing emotion regulation. Children raised by caregivers with poor parenting skills may have more difficulties to generalize adaptive coping behaviors based on with limited opportunity for trial-and-error instrumental learning and ill-adapted reinforcers. This is the first study that empirically confirms the high rate of DMDD in foster care youths who are often exposed to ACEs. Foster care youths with mood disorders also more frequently presented associated behavioral problems such as running away and substance abuse. These associations are likely due to an underlying impulsive trait (50); however, such hypotheses were not formally tested in this study. The rate of suicidal behavior and psychotic symptoms in foster care youths were not significantly different from the other groups of patients, contradicting previous findings reported in youths exposed to cumulative forms of ACEs (11, 13).

The current study confirms the high frequency of neurodevelopmental disorders in foster care youths with mood disorders compared to other patients. If previous studies put some emphasis on the high rate of ADHD in this population (1, 2), our findings found associations with intellectual development disorder, developmental coordination disorders, and communication disorders as reported earlier in the few studies that have searched for it (4). In our study, youths in the PI group were on average 2.6 times more at-risk of having a diagnosis of any neurodevelopmental disorder compared to those without child welfare intervention. These findings were consistent when comparing across the three groups the rate of perinatal risk factors, the delays in milestone psychomotor acquisition, and the difficulties in academic settings in the current research. A lack of awareness of neurodevelopmental disorders in foster care patients with mood disorders may represent a missed opportunity to provide remediation interventions, and finally to reach clinical remission and better school inclusion.

The mean level of functional impairment (based on CGAS score at admission) of inpatient youths with mood disorders living in foster care did not significantly differ from those in the NPI group and those in the WCWI group. This finding contradicts results from community-based and clinical samples (12, 14). It is possible that Berkson’s bias explains that participants in the control groups (i.e., NPI, WCWI groups) who were recruited via university clinical research programs were principally composed of patients with severe and impairing forms of mood disorders.

#### 4.1.2. Therapeutics

The higher rates of emergency care and hospitalization observed in foster care youths with mood disorders compared to other patients are consistent with the literature. As the patients’ clinical severity (based on the CGI-S) were comparable across the three groups, the overuse of emergency services is likely to be linked to other risk factors, such as an impulsive trait or low level of adhesion to care (6, 51). An assumption which is indirectly supported by our data considering the higher rate of care under constraints reported in patients in the PI group compared to the two other groups (Table 4).

Access to occupational therapy was lower among patients in the PI group compared to those in the NPI group, while the rate of a developmental coordination disorder was comparable between these two groups. Giannitelli et al. (4) showed that inpatients in foster care had on average lower access to speech therapy compared to other inpatients. The co-occurrence of more frequent neurodevelopmental disorders and less access to rehabilitation care is particularly concerning with regard to the academic difficulties faced by these patients reported here and in previous studies (36, 37).

No significant difference was found across the groups with regard to the use of antidepressants and mood stabilizers. In line with prior studies, (5, 6) the rate of antipsychotics prescription was higher in the PI group compared to the other groups. The low sample size prevents us to provide additional subgroup analyses to determine the influence of clinical indications on treatments choice. The lower proportions of “typical” forms of depression, i.e., MDD, reported in the PI group could in theory be associated with lower rates of antidepressant prescription, as no clear guidelines support the use of antidepressants in youths with PDD and DMDD, and no medication received an authorization by the national competent authorities in American or European countries for these indications (52).

Our study did not find any significant difference in the level of treatment response between the three groups, both at the clinical (i.e., CGI-I) and the functional level (i.e., change in CGAS score during the stay). These results should be interpreted with caution because these variables were collected only in settings 1 and 2, i.e., in patients referred for full-time hospitalization. It would be interesting to confirm these results in patients referred to intensive outpatient care. Although preliminary, these results support the hypothesis that the poorer response to treatment observed in foster care youths mainly involve environmental factors influencing the likelihood to access and/or to adhere to quality care treatment.

### 4.2. Strengths and limitations

These results must be considered in light of some methodological flaws that may limit their interpretation. The specific characteristics of inclusion sites should be considered when discussing the generalizability of these results. The outpatient and inpatient facilities involved mostly patients with severe treatment-refractory illness. For example, in site 1 a substantial proportion of youths could have been addressed from another hospital in a context of treatment-refractory psychiatric disorders, as the department became a specialized center for catatonic syndrome, bipolar disorder, and rare neurodevelopmental diseases with psychiatric manifestations. This secondary analysis of previous observational studies did not allow us the collection of information that may be pertinent for research purposes (e.g., patient’s perceived alliance with the clinician, borderline personality traits) or to assess treatment efficacy under blind conditions. As noted above, a lack of statistical power due to a large number of potentially confounding variables and relatively small sample size complicated the interpretation of non-significant results. One could regret that the cross-sectional design of the study precluded us from examining how placement decision impacts the trajectory of emotional symptoms. The discussion about effect of family separation as a precipitating factor for mood disorders (35) should not overlook the fact that the placement decision is in most cases an opportunity to remove the child from severe stressors involving dysfunctional family interactions.

### 4.3. Clinical and research implications

Our current report invites clinicians to put specific emphasis on associated developmental impairments that may complicate diagnosis assessment in foster care youths with mood disorders. In addition to the patient’s possible initial mistrust in mental health professionals due to trauma- and attachment-related factors, associated cognitive impairments such as attention, executive function, memory, language could complicate the assessment of mood symptoms. This may be particularly true for mood symptoms that refer to complex emotional states, such as anhedonia, guilt or shame. Of note, such cognitive impairments have been reported in young people exposed to repeated ACEs even in the absence of neurodevelopmental disorders (21, 53, 54). The assessment of perinatal risk factors influencing both neurodevelopmental and emotional disorders may be particularly hard in the context of family-separation with little reliable information about pregnancy and early childhood (5). Finally, clinicians could be prone to consider mood and neurodevelopmental disorders as differential rather than combined diagnoses (55). The identification of a combined form of mood and neurodevelopmental disorders in foster care youths is worth considering as it could represent an important therapeutic opportunity (5), such as specific remediation interventions for cognitive impairments (36).

Our results confirm data from studies conducted in the US, in particular the high rate of antipsychotic prescription (5, 6, 56, 57). We observed that foster care youths did not have a worse prognosis than other inpatients. This preliminary data supports the view that youths with mood disorders have comparable response rates to interventions provided during a hospitalization (i.e., in an environment where the youth’s access and maintenance to the interventions is largely controlled, unlike ambulatory care). Said differently, it is likely that the negative effect of ACEs on the treatment response of pediatric mood disorders (26–29) is undermined by difficulties to access and to adhere to quality interventions rather than the effect of this intervention *per se* (which explains our title). Such an assumption could be empirically tested, for example, by comparing the response rate of antidepressants in youths exposed to ACEs in per protocol and in intent-to-treat analyses. The negative effect of ACEs on the treatment response of mood disorders noted in adults (14, 25) could be partly underpinned by the effect of persisting untreated mood symptoms due to life-long barriers to care. Chronic subsyndromal emotional disturbances in these patients could influence individual (e.g., cognitive distortions and biases) and social functioning (conjugal status, academic/professional achievement) which are also identified as moderators of the effectiveness of treatments provided for mood disorders (58). Following this, emphasis must therefore be placed on the influence of individual and environmental factors influencing all steps of the care pathway of the foster care youths leading to access and to adhere to quality interventions.

## 5. Conclusion

A higher rate of chronic forms of depression, with predominant irritability, was observed in youths with mood disorders in foster care compared to other patients. We also found a high rate of associated neurodevelopmental disorders in this population, more frequent prescriptions of antipsychotics and use of emergency care. If many factors could influence the access and the adhesion to mental health interventions in foster care youths with mood disorders, we did not find any significant difference in the response to the therapeutic care provided during hospitalization. This finding is worth noting considering the usual therapeutic “defeatism” among clinicians caring for foster care youths, an assumption probably maintained by a lack of empirical evidence (16).

## Data availability statement

The original contributions presented in the study are included in the article/supplementary materials, further inquiries can be directed to the corresponding author.

## Ethics statement

The studies involving humans were approved by Autorisation CNIL MR004 N°2208336v0. The studies were conducted in accordance with the local legislation and institutional requirements. The ethics committee/institutional review board waived the requirement of written informed consent for participation from the participants or the participants' legal guardians/next of kin because the data analyzed were exclusively based on information collected during usual care.

## Author contributions

XB, J-MG, DC, and RL: study concept, design, and drafting the manuscript. J-MG, RL, XB, and HL: acquisition of data. XB, J-MG, DC, RL, HP, JW, and FE-K: interpretation of data. PG, JW, and FE-K: critical revision of the manuscript for important intellectual content. XB, HL, HP, AC, DC, RL, JR, PG, FE-K, JW, and J-MG: final draft. All authors contributed to the article and approved the submitted version.

## References

[1] Bronsard G Alessandrini M Fond G Loundou A Auquier P Tordjman S. The prevalence of mental disorders among children and adolescents in the child welfare system: a systematic review and meta-analysis. Medicine. (2016) 95:e2622. doi: 10.1097/md.0000000000002622, PMID: 26886603 PMC4998603

[2] Engler AD Sarpong KO Van Horne BS Greeley CS Keefe RJ. A systematic review of mental health disorders of children in Foster Care. Trauma Violence Abuse. (2022) 23:255–64. doi: 10.1177/1524838020941197, PMID: 32686611

[3] Cicchetti D. Socioemotional, personality, and biological development: illustrations from a multilevel developmental psychopathology perspective on child maltreatment. Annu Rev Psychol. (2016) 67:187–211. doi: 10.1146/annurev-psych-122414-033259 26726964

[4] Giannitelli M Plaza M Guillemont F Hingant A Bodeau N Chauvin D. Troubles du langage oral et écrit chez des jeunes pris en charge par l’aide sociale à l’enfance et bénéficiant de soins hospitaliers. Neuropsychiatr Enfance Adolesc. (2011) 59:492–500. doi: 10.1016/j.neurenf.2011.10.001

[5] Keeshin B Forkey HC Fouras G MacMillan HL. Children exposed to maltreatment: assessment and the role of psychotropic medication. Pediatrics. (2020) 145:3751. doi: 10.1542/peds.2019-3751 31964760

[6] Pecora PJ White CR Jackson LJ Wiggins T. Mental health of current and former recipients of foster care: a review of recent studies in the USA. Child Fam Soc Work. (2009) 14:132–46. doi: 10.1111/j.1365-2206.2009.00618.x

[7] Bellamy JL Gopalan G Traube DE. A national study of the impact of outpatient mental health services for children in long-term foster care. Clin Child Psychol Psychiatry. (2010) 15:467–79. doi: 10.1177/1359104510377720, PMID: 20923897 PMC3049724

[8] Petrenko CL Culhane SE Garrido EF Taussig HN. Do youth in out-of-home care receive recommended mental health and educational services following screening evaluations? Child Youth Serv Rev. (2011) 33:1911–8. doi: 10.1016/j.childyouth.2011.05.015, PMID: 21912444 PMC3169801

[9] Stein RE Hurlburt MS Heneghan AM Zhang J Kerker B Landsverk J. For better or worse? Change in service use by children investigated by child welfare over a decade. Acad Pediatr. (2016) 16:240–6. doi: 10.1016/j.acap.2016.01.019 26851614 PMC5560869

[10] Guedj-Bourdiau MJ Guilé JM Garny de la Rivière S Pace U Cohen D. Unmet needs and classical pitfalls in the Management of Adolescents with Behavioral Problems in emergency. Front Psych. (2021) 12:527569. doi: 10.3389/fpsyt.2021.527569, PMID: 33643084 PMC7907428

[11] Dunn EC McLaughlin KA Slopen N Rosand J Smoller JW. Developmental timing of child maltreatment and symptoms of depression and suicidal ideation in young adulthood: results from the National Longitudinal Study of adolescent health. Depress Anxiety. (2013) 30:955–64. doi: 10.1002/da.22102, PMID: 23592532 PMC3873604

[12] Holshausen K Bowie CR Harkness KL. The relation of childhood maltreatment to psychotic symptoms in adolescents and young adults with depression. J Clin Child Adolesc Psychol. (2016) 45:241–7. doi: 10.1080/15374416.2014.952010 25411823

[13] King CA Merchant CR. Social and interpersonal factors relating to adolescent suicidality: a review of the literature. Arch Suicide Res. (2008) 12:181–96. doi: 10.1080/13811110802101203, PMID: 18576200 PMC2989173

[14] Nanni V Uher R Danese A. Childhood maltreatment predicts unfavorable course of illness and treatment outcome in depression: a meta-analysis. Am J Psychiatry. (2012) 169:141–51. doi: 10.1176/appi.ajp.2011.11020335, PMID: 22420036

[15] Benarous X Consoli A Milhiet V Cohen D. Early interventions for youths at high risk for bipolar disorder: a developmental approach. Eur Child Adolesc Psychiatry. (2016) 25:217–33. doi: 10.1007/s00787-015-0773-6 26395448

[16] Forkey H Szilagyi M. Foster care and healing from complex childhood trauma. Pediatr Clin N Am. (2014) 61:1059–72. doi: 10.1016/j.pcl.2014.06.015, PMID: 25242716

[17] Tarren-Sweeney M. The mental health of children in out-of-home care. Curr Opin Psychiatry. (2008) 21:345–9. doi: 10.1097/YCO.0b013e32830321fa 18520738

[18] Consoli A Cohen D. Manic-like symptoms in youths: diagnosis issues and controversies. Neuropsychiatr Enfance Adolesc. (2013) 61:154–9. doi: 10.1016/j.neurenf.2012.10.002

[19] Griffin G McClelland G Holzberg M Stolbach B Maj N Kisiel C. Addressing the impact of trauma before diagnosing mental illness in child welfare. Child Welfare. (2011) 90:69–89. PMID: 22533043

[20] Teicher MH Samson JA. Childhood maltreatment and psychopathology: a case for ecophenotypic variants as clinically and neurobiologically distinct subtypes. Am J Psychiatry. (2013) 170:1114–33. doi: 10.1176/appi.ajp.2013.12070957 23982148 PMC3928064

[21] Cohen D. Traumatismes et traces: données expérimentales. Neuropsychiatr Enfance Adolesc. (2012) 60:315–23. doi: 10.1016/j.neurenf.2011.09.005

[22] Anderson HD. Suicide ideation, depressive symptoms, and out-of-home placement among youth in the U.S. child welfare system. J Clin Child Adolesc Psychol. (2011) 40:790–6. doi: 10.1080/15374416.2011.614588, PMID: 22023270

[23] Munson MR McMillen C. Trajectories of depression symptoms among older youths exiting Foster Care. Soc Work Res. (2010) 34:235–49. doi: 10.1093/swr/34.4.235, PMID: 25076833 PMC4112472

[24] Valdez CE Bailey BE Santuzzi AM Lilly MM. Trajectories of depressive symptoms in foster youth transitioning into adulthood: the roles of emotion dysregulation and PTSD. Child Maltreat. (2014) 19:209–18. doi: 10.1177/1077559514551945 25248919

[25] Agnew-Blais J Danese A. Childhood maltreatment and unfavourable clinical outcomes in bipolar disorder: a systematic review and meta-analysis. Lancet Psychiatry. (2016) 3:342–9. doi: 10.1016/s2215-0366(15)00544-1, PMID: 26873185

[26] Asarnow JR Emslie G Clarke G Wagner KD Spirito A Vitiello B. Treatment of selective serotonin reuptake inhibitor-resistant depression in adolescents: predictors and moderators of treatment response. J Am Acad Child Adolesc Psychiatry. (2009) 48:330–9. doi: 10.1097/chi.0b013e3181977476, PMID: 19182688 PMC2754157

[27] Emslie GJ Kennard BD Mayes TL. Predictors of treatment response in adolescent depression. Pediatr Ann. (2011) 40:300–6. doi: 10.3928/00904481-20110512-05 21678888

[28] Lewis CC Simons AD Nguyen LJ Murakami JL Reid MW Silva SG. Impact of childhood trauma on treatment outcome in the treatment for adolescents with depression study (TADS). J Am Acad Child Adolesc Psychiatry. (2010) 49:132–40. doi: 10.1016/J.JAAC.2009.10.007 PMID: 20215935

[29] Shamseddeen W Asarnow JR Clarke G Vitiello B Wagner KD Birmaher B. Impact of physical and sexual abuse on treatment response in the treatment of resistant depression in adolescent study (TORDIA). J Am Acad Child Adolesc Psychiatry. (2011) 50:293–301. doi: 10.1016/j.jaac.2010.11.019, PMID: 21334569 PMC3073648

[30] Benarous X Raffin M Bodeau N Dhossche D Cohen D Consoli A. Adverse childhood experiences among inpatient youths with severe and early-onset psychiatric disorders: prevalence and clinical correlates. Child Psychiatry Hum Dev. (2017b) 48:248–59. doi: 10.1007/s10578-016-0637-4, PMID: 27002816

[31] Serim Demirgoren B Ozbek A Gencer O. Factors affecting improvement of children and adolescents who were treated in the child and adolescent psychiatry inpatient unit. J Int Med Res. (2017) 45:1318–23. doi: 10.1177/0300060517713833, PMID: 28606027 PMC5625538

[32] Benarous X Cravero C Jakubowicz B Morales P Cohen D. Looking for the good timing: predictors of length of stay and therapeutic outcomes in adolescent inpatients with severe or treatment-refractory mood disorders. J Child Adolesc Psychopharmacol. (2021a) 31:268–78. doi: 10.1089/cap.2020.0138 33909453

[33] Setoya Y Saito K Kasahara M Watanabe K Kodaira M Usami M. Evaluating outcomes of the child and adolescent psychiatric unit: a prospective study. Int J Ment Health Syst. (2011) 5:7. doi: 10.1186/1752-4458-5-7, PMID: 21453481 PMC3079682

[34] Barbe RP Bridge JA Birmaher B Kolko DJ Brent DA. Lifetime history of sexual abuse, clinical presentation, and outcome in a clinical trial for adolescent depression. J Clin Psychiatry. (2004) 65:77–83. doi: 10.4088/jcp.v65n0113, PMID: 14744173

[35] Mitchell M. “No One Acknowledged My Loss and Hurt”: Non-death Loss, Grief, and Trauma in Foster Care. Child Adolesc. Soc. Work J. (2018) 35. doi: 10.1007/s10560-017-0502-8

[36] Männistö II Pirttimaa RA. A review of interventions to support the educational attainments of children and adolescents in foster care. Adopt Fostering. (2018) 42:266–81. doi: 10.1177/0308575918791627

[37] Zima BT Bussing R Freeman S Yang X Belin TR Forness SR. Behavior problems, academic skill delays and school failure among school-aged children in Foster Care: their relationship to placement characteristics. J Child Fam Stud. (2000) 9:87–103. doi: 10.1023/A:1009415800475

[38] Benarous X Iancu C Guilé JM Consoli A Cohen D. Missing the forest for the trees? A high rate of motor and language impairments in disruptive mood dysregulation disorder in a chart review of inpatient adolescents. Eur Child Adolesc Psychiatry. (2021b) 30:1579–90. doi: 10.1007/s00787-020-01636-y 32918099

[39] Benarous X Renaud J Breton JJ Cohen D Labelle R Guilé JM. Are youths with disruptive mood dysregulation disorder different from youths with major depressive disorder or persistent depressive disorder? J Affect Disord. (2020) 265:207–15. doi: 10.1016/j.jad.2020.01.020 32090743

[40] Cohen D Hanin C Benarous X. Debate: developmental and integrative approaches in child and adolescent psychiatry inpatient facilities: the case of a tertiary university hospital in Paris. Child Adolesc Ment Health. (2021) 26:171–3. doi: 10.1111/camh.12461, PMID: 33779120

[41] Boudjerida A. Labelle R Bergeron L Berthiaume C Guilé JM Breton JJ. Development and Initial Validation of the Disruptive Mood Dysregulation Disorder Questionnaire Among Adolescents From Clinic Settings. Front Psychiatry. (2022) 13:617991. doi: 10.3389/fpsyt.2022.617991 35250652 PMC8891213

[42] Breton JJ Labelle R Huynh C Berthiaume C St-Georges M Guile JM. Clinical characteristics of depressed youths in child psychiatry. J Can Acad Child Adolesc Psychiatry. (2012) 21:16–29. 22299011 PMC3269244

[43] Marchand WR Wirth L Simon C. Adverse life events and pediatric bipolar disorder in a community mental health setting. Community Ment Health J. (2005) 41:67–75. doi: 10.1007/s10597-005-2600-x, PMID: 15932053

[44] Garno JL Goldberg JF Ramirez PM Ritzler BA. Impact of childhood abuse on the clinical course of bipolar disorder. Br J Psychiatry. (2005) 186:121–5. doi: 10.1192/bjp.186.2.121 15684234

[45] Kaufman J Birmaher B Brent D Rao U Flynn C Moreci P. Schedule for affective disorders and schizophrenia for school-age children-present and lifetime version (K-SADS-PL): initial reliability and validity data. J Am Acad Child Adolesc Psychiatry. (1997) 36:980–8. doi: 10.1097/00004583-199707000-00021 9204677

[46] Busner J Targum SD. The clinical global impressions scale: applying a research tool in clinical practice. Psychiatry. (2007) 4:28–37. PMID: 20526405 PMC2880930

[47] Jones SH Thornicroft G Coffey M Dunn G. A brief mental health outcome scale-reliability and validity of the global assessment of functioning (GAF). Br J Psychiatry. (1995) 166:654–9. doi: 10.1192/bjp.166.5.654 7620753

[48] Dvir Y Ford JD Hill M Frazier JA. Childhood maltreatment, emotional dysregulation, and psychiatric comorbidities. Harv Rev Psychiatry. (2014) 22:149–61. doi: 10.1097/HRP.0000000000000014, PMID: 24704784 PMC4091823

[49] Brotman MA Kircanski K Stringaris A Pine DS Leibenluft E. Irritability in youths: a translational model. Am J Psychiatry. (2017) 174:520–32. doi: 10.1176/appi.ajp.2016.16070839 28103715 PMC13335380

[50] Leloux-Opmeer H Kuiper C Swaab H Scholte E. Characteristics of children in Foster Care, family-style group care, and residential care: a scoping review. J Child Fam Stud. (2016) 25:2357–71. doi: 10.1007/s10826-016-0418-5 27440989 PMC4933723

[51] Turney K Wildeman C. Mental and physical health of children in Foster Care. Pediatrics. (2016) 138:1118. doi: 10.1542/peds.2016-1118 27940775

[52] Benarous X Consoli A Guile JM Garny de La Riviere S Cohen D Olliac B. Evidence-based treatments for youths with severely dysregulated mood: a qualitative systematic review of trials for SMD and DMDD. Eur Child Adolesc Psychiatry. (2017a) 26:5–23. doi: 10.1007/s00787-016-0907-5, PMID: 27662894

[53] Benarous X Guile JM Consoli A Cohen D. A systematic review of the evidence for impaired cognitive theory of mind in maltreated children. Front Psych. (2015) 6:108. doi: 10.3389/fpsyt.2015.00108, PMID: 26283975 PMC4516890

[54] Kaufman J Gelernter J Hudziak JJ Tyrka AR Coplan JD. The research domain criteria (RDoC) project and studies of risk and resilience in maltreated children. J Am Acad Child Adolesc Psychiatry. (2015) 54:617–25. doi: 10.1016/j.jaac.2015.06.001, PMID: 26210330 PMC4515569

[55] Szymanski K Sapanski L Conway F. Trauma and ADHD–association or diagnostic confusion? A clinical perspective. J Infant, Child, Adol Psychother. (2011) 10:51–9. doi: 10.1080/15289168.2011.575704

[56] Mackie TI Hyde J Palinkas LA Niemi E Leslie LK. Fostering psychotropic medication oversight for children in Foster Care: a National Examination of States’ monitoring mechanisms. Admin Pol Ment Health. (2017) 44:243–57. doi: 10.1007/s10488-016-0721-x 26860953

[57] Tan C Greiner MV Nause K Shahabuddin Z Beal SJ. Mental health diagnoses, health care utilization, and placement stability on antipsychotic prescribing among Foster Care youth. Acad Pediatr. (2022) 23:675–80. doi: 10.1016/j.acap.2022.08.005 36031052

[58] Papakostas GI Fava M. Predictors, moderators, and mediators (correlates) of treatment outcome in major depressive disorder. Dialogues Clin Neurosci. (2008) 10:439–51. doi: 10.31887/DCNS.2008.10.4/gipapakostas, PMID: 19170401 PMC3181892

